# Characterization of scientific studies usually cited as evidence of adverse effects of GM food/feed

**DOI:** 10.1111/pbi.12798

**Published:** 2017-08-16

**Authors:** Miguel A. Sánchez, Wayne A. Parrott

**Affiliations:** ^1^ Asociación Gremial ChileBio CropLife Santiago Chile; ^2^ Department of Crop and Soil Sciences University of Georgia Athens GA USA

**Keywords:** GMO, GM food/feed safety, scientific studies, evidence

## Abstract

GM crops are the most studied crops in history. Approximately 5% of the safety studies on them show adverse effects that are a cause for concern and tend to be featured in media reports. Although these reports are based on just a handful of GM events, they are used to cast doubt on all GM crops. Furthermore, they tend to come from just a few laboratories and are published in less important journals. Importantly, a close examination of these reports invariably shows methodological flaws that invalidate any conclusions of adverse effects. Twenty years after commercial cultivation of GM crops began, a bona fide report of an adverse health effect due to a commercialized modification in a crop has yet to be reported.

## Introduction

Concerns over the lack of safety of genetically modified (GM) crops are used as arguments to ban them, or at least to regulate them heavily. Many citizens, politicians and countries generally take those arguments to reinforce misconceptions about GM crops. Anecdotal and newspaper reports also reinforce an anti‐GM stance (Wunderlich and Gatto, 2015). Usually, the debate is driven by political and ideological arguments instead of science‐based discussion (Trewavas and Leaver, 2001). Some aspects of agriculture in general, such as herbicides, monocultures and intellectual property, also contribute to concerns over GM crops (Hilbeck *et al*., 2015), despite applying equally to conventional agriculture.

Although referred to monolithically as GM crops in conversation, GM crops are quite distinct from each other; the only thing in common they share with each other is the process—recombinant DNA—used to produce them. Each individual use of recombinant DNA to produce a GM crop is known as an ‘event’. In the United States, the FDA has reviewed 153 events to date (http://www.accessdata.fda.gov/scripts/fdcc/?set=Biocon), many of which are on the market. Yet, most of the publications that are recurrently cited as evidence against GM crop safety (Domingo and Bordonaba, 2011; Dona and Arvanitoyannis, 2009; Hilbeck *et al*., 2015; Magaña‐Gómez and de la Barca, 2009; Seralini 2011) centre on a small number of studies on specific events and that received extensive media coverage. The hundreds of scientific studies that do not support safety concerns go unnoticed in the public debate (Nicolia *et al*., 2014).

What follows is an assessment of original research papers addressing food/feed safety aspects of GM crops, which are used frequently as evidence of adverse effects and health risks of GM crops. Potential conflict of interests (COI), the scientific quality of the studies and the logic and credibility of arguments and conclusions were appraised. To the best of our knowledge, this is the first paper analysing this information all at once.

## About the papers evaluated

The papers selected for study include those cited in four reviews of adverse effects of GM crops (Domingo and Bordonaba, 2011; Dona and Arvanitoyannis, 2009; Magaña‐Gómez and de la Barca, 2009; Seralini 2011). References cited in the Internet by GM Free USA, Coalition for a GM Free India and GM Watch were added. Studies not published in scientific journals (e.g. Ermakova, Velimirov, Surov, abstracts) as well as those reports evaluating only immunogenicity of pure proteins, instead of whole food or GM crops studies, were not included. Reports in a language other than English were not considered.

Thirty‐five articles meet the above criteria (Table 1). These 35 studies represent fewer than 5% of all published studies assessing GM food/feed safety (Nicolia *et al*., 2014; Sánchez, 2015).

**Table 1:** Papers cited frequently as evidence of adverse effects of GE or specific GM crops on health

Main author	Year	Journal	IF	Crop	Trait	Event	Model	Claimed impact health	Main shortcomings
Abdo *et al*.	2014	Food Nutr Sci	ni	Maize	IR	MON810	Rat	Hepatic alterations	No nutritional analysis of diet; no measure of mycotoxin content; no information on crop source
Ayyadurai & Deonikar	2015	Agric Sci	ni	Soya	HT	nm	*in silico*	Accumulation of formaldehyde; depletion of glutathione	No data supporting the study
Battistelli *et al*.	2008	Microscopie	ni	Soya	HT	40‐3‐2	Mouse	Pancreatic alterations	No nutritional analysis of diet; isoflavone content not measured; no information about varieties used
Battistelli *et al*.	2010	Eur J Histochem	1.809	Soya	HT	40‐3‐2	Mouse	Intestinal alterations	No nutritional analysis of diet; isoflavone content not measured; no information about varieties used
Brasil *et al*.	2009	Anat Rec (Hoboken)	1.490	Soya	nm	nm	Rat	Ovary and uterus alterations	No nutritional analysis of diet; no information on crop source
Carman *et al*.	2013	J Org Sys	ni	Maize; Soya	IR; HT	NK603; MON863; MON810; 40‐3‐2	Pig	Higher rate of severe stomach inflammation; thicker uterus	Flawed statistics; no nutritional analysis of diet
Cisterna *et al*.	2008	Eur J Histochem	1.629	Soya	HT	40‐3‐2	Mouse	Decrease of pre‐mRNA transcription in 2‐, 4‐, 8‐cell embryos	No nutritional analysis of diet; isoflavones content not measured; no information about varieties used
de Vendomois *et al*.	2009	Int J Biol Sci	2.865	Maize	HT; IR	NK603; MON863; MON810	Rat	Hepatorenal alterations	Flawed statistics (fishing for significance)
El‐Kholy *et al*.	2014	Nutrients	3.270	Soya	nm	nm	Rat	Increase in serum level of lipid peroxidation; decrease in glutathione transferase; cytogenicity	No use of non‐GM soya bean as control
El‐Shamei *et al*.	2012	J Am Sci	ni	Maize	IR	MON810	Rat	Histopathological changes of liver, kidney, testis, spleen and small intestine	No nutritional analysis of diet; no measure of mycotoxin content; no information on crop source
Ewen & Pusztai	1999	Lancet	10.197	Potato	IR	nc	Rat	Differences in the thickness of the gut epithelium	Inadequate sample size; protein‐deficient diets; no dose–response studies; lack of proper controls
Fares & El‐Sayed	1998	Nat Toxins	0.691	Potato	IR	nc	Mouse	Intestinal alterations	Cause and effect not established
Finamore *et al*.	2008	J Agr Food Chem	2.562	Maize	IR	MON810	Mouse	Immune response	Mycotoxins above maximum allowed (food standards)
Gab‐Alla *et al*.	2012	J Am Sci	ni	Maize	IR	MON810	Rat	Differences in organs/body weight; changes in serum biochemistry	No nutritional analysis of diet; no measure of mycotoxin content; no information on crop source
Ibrahim & Okasha	2016	Exp Toxicol Pathol	1.716	Maize	IR	MON810	Rat	Histopathological changes of small intestine	No nutritional analysis of diet; no measure of mycotoxin content; no information on crop source
Kiliç & Akay	2008	Food Chem Toxicol	2.321	Maize	IR	nm	Rat	Hepatorenal alterations	No biological relevance (no health concerns after three generations)
Kiliçgün *et al*.	2013	J Clin Anal Med	ni	Maize	IR	nm	Rat	Alterations in length, height and weight of organs; alterations in haematology values	No nutritional analysis of diet; no measure of mycotoxin content; no information on crop source.
Magaña‐Gómez *et al*.	2008	J Appl Toxicol	2.127	Soya	HT	40‐3‐2	Mouse	Pancreatic alterations	No information on crop source and varieties used
Malatesta *et al*.	2002	Cell Struct Funct	0.872	Soya	HT	40‐3‐2	Mouse	Hepatic alterations	No nutritional analysis of diet; isoflavone content not measured; no information about varieties used
Malatesta *et al*.	2002	J Anat	1.660	Soya	HT	40‐3‐2	Mouse	Pancreatic alterations	No nutritional analysis of diet; isoflavone content not measured; no information about varieties used
Malatesta *et al*.	2003	Eur J Histochem	1.041	Soya	HT	40‐3‐2	Mouse	Pancreatic alterations	No nutritional analysis of diet; isoflavone content not measured; no information about varieties used
Malatesta *et al*.	2005	Eur J Histochem	0.990	Soya	HT	40‐3‐2	Mouse	Hepatic alterations	No nutritional analysis of diet; isoflavone content not measured; no information about varieties used
Malatesta *et al*.	2008	Histochem Cell Biol	2.320	Soya	HT	40‐3‐2	Mouse	Hepatic alterations	No nutritional analysis of diet; isoflavone content not measured; no information about varieties used.
Oraby *et al*.	2015	Turk J Biol	1.343	Maize; Soya	nm	nm	Rat	Genotoxicity in germ and liver cells	Different control diet (wheat)
Prescott *et al*.	2005	J Agr Food Chem	2.507	Pea	IR	nc	Mouse	Immune response	Results are not specific to biotechnology
Sagstad *et al*.	2007	J Fish Dis	1.712	Maize	IR	MON810	Salmon	Immune response	Presence of mycotoxins in the experimental diet
Séralini *et al*.	2007	Arch Environ Con Tox	1.620	Maize	IR	MON863	Rat	Hepatorenal alterations	Flawed statistics (fishing for significance)
Séralini *et al*.	2014	Env Sci Eur	ni	Maize	HT	NK603	Rat	Hepatorenal alterations; tumour rate altered	Nonappropriate animal model; flawed statistics; inadequate sample size
Trabalza‐Marinucci *et al*.	2008	Livest Sci	1.091	Maize	IR	BT176	Sheep	Hyperplasia of ruminal epithelial basal cells; higher immune response to S. abortus ovis	No measure of mycotoxin content; no information on crop source
Tudisco *et al*.	2006	Anim Sci	1.021	Soya	HT	40‐3‐2	Rabbit	Lactic dehydrogenase increased in kidney and heart	Possible plagiarism; no biological relevance
Tudisco *et al*.	2007	Ital J Anim Sci	0.218	Soya	HT	40‐3‐2	Goat	Transgene fragments detected in tissues	No biological relevance
Tudisco *et al*.	2010	Animal	1.721	Soya	HT	40‐3‐2	Goat	Transgene fragments detected in tissues; lactic dehydrogenase increased	Retracted because of plagiarism; no biological relevance; no information on crop source
Tudisco *et al*.	2015	Small Rum Res	1.125	Soya	HT	40‐3‐2	Goat	Decrease in growth performances of goats; transgene fragments detected in colostrum	Possible plagiarism; no information on crop source; no information about varieties used
Vecchio *et al*.	2004	Eur J Histochem	0.845	Soya	HT	40‐3‐2	Mouse	Testis alterations	No nutritional analysis of diet; isoflavone content not measured; no information about varieties used
Yum *et al*.	2005	Allergy Asthma Proc	0.733	Soya	nm	nm	Human	Allergy (not according to the authors)	No information on crop source; inadequate sample size

ni, not indexed; nm, not mentioned; nc, noncommercial; IF, impact factor (year of publication).

In the 35 articles assessed, the herbicide‐tolerant soya bean event 40‐3‐2 has been the GM crop/feed most analysed, for a total of 15 studies (43%). The insect‐resistant maize event, MON810, is part of eight studies (23%). Another three (9%) evaluated noncommercial events, two on GM potato and one on GM pea. Seven studies (20%) do not indicate which event was evaluated, which makes it impossible to replicate the experiments or interpret the results. Maize NK603, MON863 and BT176 are the other tested events. All of these were among the first events to reach the market, or at least, to appear in the literature.

**Figure 1:**
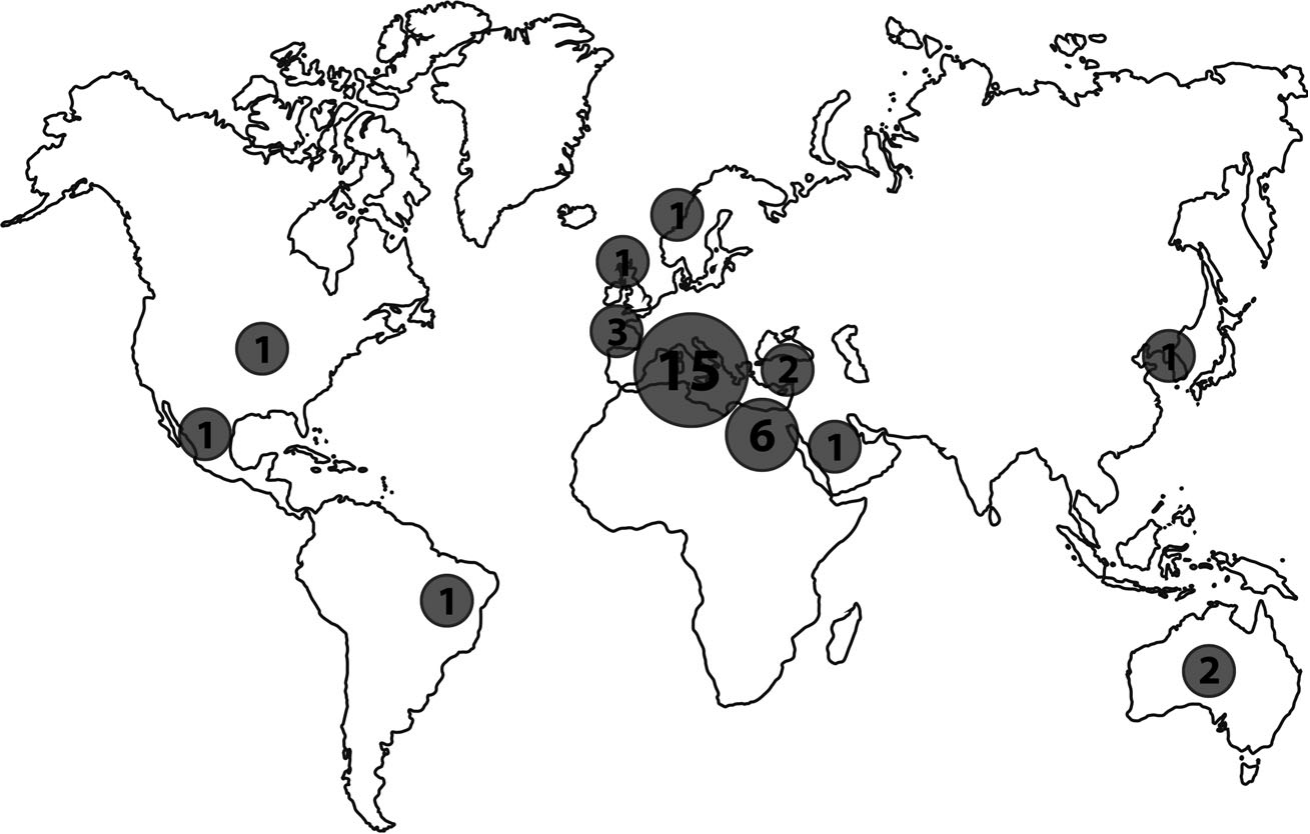
Geographical distribution of origin of papers cited frequently as evidence of adverse effects of GE or specific GM crops on health.

The geographical origin of the 35 studies is striking. There are just a few laboratories from a few places that are responsible for articles claiming adverse effects. While 57% (20) were conducted in Europe, 43% (15) were carried out in Italy (Figure 1). Egyptian scientists contributed 17% (6) of the studies here assessed. Only one paper reviewed here, though written in Australia, is based on research conducted in the USA.

The most frequent author on these publications, having co‐authored 11 of 35 studies (31%), is the Malatesta group at the University of Urbino and University of Verona, Italy. Nine of their articles are on soya bean 40‐3‐2 and represent 60% of all studies suggesting adverse effects from this event. Their studies on maize events BT176 and NK603 were co‐authored with the M. Trabalza‐Marinucci (Università degli Studi di Perugia, Italy) and G‐E. Séralini (University of Caen, France) teams, respectively. R. Tudisco and F. Infascelli (both from University of Naples Federico II) were part of four studies (11%), all of which assessed soya bean 40‐3‐2. Altogether, 87% of all studies on event 40‐3‐2 come from the Malatesta and Infascelli groups. G‐E. Séralini was the corresponding author in three studies (9%) on different events of GM maize, corresponding to 25% of all studies assessing GM maize.

Because of the relevance of food safety, any well‐conducted study under rigorous standards of scientific quality and showing adverse effects of any GM food/crop could and would be published in the most prominent journals. However, most studies often used in the public debate against GM food/crops have been published in journals with lower visibility; eight were even published in journals without a listed impact factor (Table 1).

The only study published in a high‐ranking journal has been that of Ewen & Pusztai (1999) (see Table 1). However, something usually not mentioned in the public debate is that editors published an accompanying analysis highlighting the study's flaws in many aspects of design, execution and analysis (Kuiper *et al*., 1999). Furthermore, the editor, Richard Horton, stated that publication of Ewen and Pusztai's findings was not a ‘vindication’ of Pusztai's claims. Horton argued if the study was not published, a critical evaluation of the results could not be conducted. He also cited a reviewer pointing out that he ‘would like to see the work published in the public domain so that fellow scientists can judge for themselves… if the paper is not published, it will be claimed there is a conspiracy to suppress information’ (Horton, 1999).

## Conflicts of interest

All 35 studies declared no competing interests. Financial COIs arise when research is fully or partially funded by a party with a stake in the development of GM crops or in activities anti‐GMO, whereas professional COIs arise when at least one author is affiliated with a company developing GM crops or anti‐GMO institutions, even if the research is supported through public funding. Upon our examination, fewer than half—14 of 35 (40%)—show no financial or professional COIs. It is worth noting that in the most of the cases, conflicts cannot be discerned unless an author mentions if he or she is affiliated with declared anti‐GMO institutions. The proportion of these 35 studies truly without a COI is somewhat lower than that for the vast majority of scientific studies supporting the safety of GM crops food/feed, where at least 406 of 698 reports (58.3%) have no financial or professional COIs (Sanchez 2015).

Overall, research for which the authors did not provide funding information represents 49% of the total reports (17 articles). Four of 35 articles (11%) had COIs either in terms of the author affiliation or funding source. The three studies from Seralini's group were supported by the Committee of Independent Research and Information on Genetic Engineering (CRIIGEN), which is financed by the Charles Léopold Mayer Foundation for the Progress of Humankind (FPH). This foundation has publicly supported anti‐GMOs initiatives like Inf'OGM, Foundation Sciences Citoyennes; the European Network of Scientists for Social and Environmental Responsibility (ENSSER), Combat Monsanto and Stop OGM, among others (http://alerte-environnement.fr/2012/11/12/etude-anti-ogm-de-saralini-les-petits-soldats-de-la-fondation-pour-le-progres-de-lhomme/). Likewise, Greenpeace partially funded the two studies (Séralini *et al*., 2007; de Vendomois *et al*., 2009) that found hepatorenal effects.

Although not considered in the analysis, it is worth noting that conflict of interest may have been involved in the review process of the retracted and criticized Séralini *et al*. (2012) study. J.L. Domingo, author of another review here assessed (Domingo and Bordonaba, 2011), was an editor of *Food and Chemical Toxicology* when the Seralini study was accepted.

Carman *et al*. (2013) also present COIs. George Kailis, an organic food entrepreneur having a cautionary approach to GMO (http://www.farmweekly.com.au/news/agriculture/agribusiness/general-news/technology-must-benefit-the-consumer-kailis/10451.aspx), partially funded the study. Furthermore, Verity Farms, another funder, has a non‐GMO grain‐marketing venture in the USA, and it is catalogued in the Non‐GMO Sourcebook, which is a directory of non‐GM food and agricultural products (http://www.nongmosourcebook.com/non-gmosourcebook/non-gmo-company.php?company=Verity+Farms). Plus, assistance is acknowledged from John Fagan, Arpad Puzstai and Jeffrey Smith, among others, three recognized opponents of GMO.

## Scientific quality of the studies

In general terms, all papers analysed here violate at least one of the basic standards for assessment of GM food/feed safety (Bartholomaeus *et al*., 2013; European Food Safety Authority (EFSA), 2011; ILSI 2008, 2004; Kuiper *et al*., 2001; Codex Alimentarius Commission (www.codexalimentarius.org)):

1. The control and experimental varieties should be isogenic and should have the same origin (i.e. grown in the same field, under similar conditions and in the same season) to diminish differences in nutritional content of control and experimental diets;
2. A proper statistical test should be selected before the study and not change it for convenience throughout the experiments. Furthermore, statistically significant differences are not necessarily biologically significant due to the normal range of physiological parameters among the organisms of the same species; and
3. If some differences or alterations are observed in a well‐designed study assessing GM diets, then it is necessary to contrast it with similar previous studies—if they exist—that do not show the same effects. The discrepancy should be addressed with a plausible hypothesis explaining the causes of the nonreproducibility.

Three of 35 studies (9%) did not do any experimentation either and report results solely based on statistical re‐analysis of previously published data. Séralini *et al*. (2007) and de Vendomois *et al*. (2009) suggest hepatorenal effects on rats fed GM maize. According to the authors, the use of standard statistics does not show significant changes; rather, the parameters fell within the normal range for control animals. Therefore, the authors used a nonconventional statistical method to show significant effects at low doses but not at higher doses of exposure to GM maize. These findings do not fit the standard dose–response expected in toxicology (Wilson *et al*., 2001), a fact that was dismissed by the authors, who stated they ‘considered equally important effects that were neither time nor dose related’.

Likewise, Ayyadurai and Deonikar (2015) claim that algorithms developed by them predict that GM soya bean has increased formaldehyde and decreased glutathione relative to non‐GM soya bean. Thus, they concluded that current safety assessment for GM food/crops is not adequate, given that formaldehyde has not been detected nor evaluated so far. The authors never validated their formulas by comparing GM and non‐GM soya beans for their formaldehyde content. Instead, the authors developed their formulas by searching ‘online databases including PubMed and Google Scholar’, and data from over six thousand studies were included, but the origin of the data and their validity are unknown (European Food Safety Authority (EFSA), 2015).

The Malatesta group has been involved in nine of 35 studies (26%). This series of studies is full of methodological flaws (Table 1), but perhaps the critical point is that the level of isoflavones in the soya bean diets was never measured. Such measurements are essential because these molecules can modulate the physiology of mammals due to the similarity they have with female sexual hormones (Brown and Setchell, 2001; Thigpen *et al*., 2004) and are known to be highly variable between soya bean varieties and locations (Eldridge and Kwolek, 1983).

Four studies (11%) belong to Professor Infascelli's group. Recently, Infascelli's group was informed that two papers from his group (Tudisco *et al*. 2010 and Mastellone *et al*., 2013, not assessed here) have been retracted. The articles intended to show that GM feed is detectable as GM DNA in goat kids and that these have abnormal gamma‐glutamyl transferase activity. The University of Naples Federico II, where the studies took place, conducted its own investigation and reprimanded the authors (http://napoli.repubblica.it/cronaca/2016/02/09/news/universita_-133079638/?refresh_ce%3C/a). It concluded that multiple image heterogeneities were likely attributable to digital manipulation, raising serious doubts about the reliability of the findings. The retraction notice that was posted by the journal for Mastellone *et al*. (2013) specifically cites fraud. Additional details on these publications are posted on PubPeer at https://pubpeer.com/search?q=tudisco&sessionid=B66D1BF6E6EDF5224777&commit=Search+Publications (Accessed 12 March 2017).

Finamore *et al*. (2008) reported that ingestion of GM maize provokes an immune response in mice. Although mycotoxins can affect the immune system (Sobrova *et al*., 2010), the authors dismissed a mycotoxin effect arguing the levels were modest and were only slightly higher than the maximum allowable concentration. In reality, the amount was twofold (1300 vs. 750 μg/kg) higher than allowed for deoxynivalenol.

Trabalza‐Marinucci *et al*. (2008) provide even fewer details. The authors did not mention the variety of maize used as the control, nor its origin, and mycotoxins are not addressed. In Magaña‐Gómez *et al*. (2008), it is unclear which soya bean varieties were used and whether they were isogenic lines. The origin of the soya bean tested is not mentioned, making it impossible to evaluate inherent variability that can affect results.

Carman *et al*. (2013) published on pigs fed GM or non‐GM diets for 22.7 weeks. There were no differences in feed intake, weight gain, mortality and routine blood biochemistry measurements. The GM diet was associated with severe stomach inflammation and thicker uteruses. Despite what was claimed, stomach inflammation was not tested histologically. Instead, a visual scoring of the colour of the lining of the stomach was performed, and redness was considered inflammation. It is not evident how pigs with severe stomach inflammation could have had the same weight gain as pigs with no stomach inflammation. The pattern of inflammation is likewise difficult to explain. There were more pigs with mild and moderate inflammation eating the non‐GM feed than GM feed. Further, there were fewer pigs with nil inflammation in non‐GM feed than GM feed. In addition, although the authors classified the stomach inflammation into four visual categories, they grouped them in two for statistical analyses: severe inflammation versus nonsevere inflammation.

Uteruses of non‐GM and GM‐fed pigs accounted for 0.10% and 0.12% of body weight, respectively. The heaviest uterus in the GM‐fed group weighed less than the heaviest uterus in the non‐GM‐fed group. The differences in uterus weights disappear if conventional statistical analysis is used (http://www.inexactchange.org/blog/2013/06/19/gmo-pig-study/). Finally, the authors did not provide information on the varieties of corn and soya bean used as control, and no chemical analysis of the diet was conducted (e.g. isoflavone content is unknown) to ensure the treated groups received equivalent diets.

Séralini *et al*. (2014), which is a retracted and republished article (Séralini *et al*., 2012), is one of the emblematic cases reporting criticized experiments. The authors claimed animals fed either GM maize or herbicide had higher tumour and mortality rates, as well as severe kidney and liver alterations. Among the most notorious flaws according to Arjó *et al*., 2013 are as follows: (i) the tumour rate reported is within the normal tumour rate for rats Sprague Dawley; (ii) inappropriate use of statistics: when the statistics are corrected for multiple comparisons, the negative effects disappear; (iii) inadequate sample size: that is, 10 rats per group instead of 20 rats for chemical toxicity studies, 50 rats for carcinogenicity studies and 65 rats if the survival of them is less than 50% at 104 weeks; (iv) no dose–response relationship as is expected in toxicology; (v) biased results: that is, paper contained pictures of treated rats with huge tumours, but no pictures of control group rats which had the same tumours; and (vi) the results go against an overwhelming body of evidence to the contrary.

There are several cases that do not need an extensive analysis. For instance, Oraby *et al*. (2015) reported health hazards linked to the ingestion of diets containing GM maize and GM soya bean. The control animals were not treated equally and instead were fed a diet of wheat.

El‐Kholy *et al*. (2014) investigated the effect of extra virgin olive oil and GM soya bean in rodents. However, the study aimed to compare only olive and soya bean, instead of GM and non‐GM soya bean, so no conclusions are possible.

Brasil *et al*. (2009) designed a study to compare the effects of a prolonged use of organic and GM soya bean on the lipid profile and the ovary and uterus morphology of rats. Both diets improved the lipid profile and reduced body weight, but alterations in uterine and ovarian morphology were found in animals with prolonged exposure to these diets. Although there were no differences in isoflavone content, a chemical analysis of diets was not conducted, and no information on how the crops were grown was provided. For that reason, the authors remark that small differences in diets (fat, sugar and especially protein or amino acid content) could have led to the slight differences seen between animals.

Ibrahim and Okasha (2016) evaluated the effect of GM maize on the histological structure of jejunal mucosa of adult male albino rats using different histological, immunohistochemical and morphometrical methods. Histopathological changes were claimed in the intestine. These changes are the author's interpretation of the photographs, not the interpretation of a panel of experts. No analysis of the diet was provided, so the differences, if they are real, may be due to variations in nutrients and protein content instead of GM maize per se. Furthermore, no mycotoxin content was conducted and nothing is mentioned about the origin of crops, that is no information on how the crops were grown or whether pesticides were used.

Kiliçgün *et al*. (2013) observed differences in organ size and other parameters in rats fed a diet with GM maize versus controls. In this case, the same mistakes in Ibrahim and Okasha (2016) committed (discussed above) were made. El‐Shamei *et al*. (2012) showed that rats fed on GM maize showed histopathological changes in liver, kidney, testis, spleen and small intestine, again without being interpreted by a panel of experts. Aside from not indicating the number of animals that were or not affected in the different conditions, no mycotoxin content was conducted, and nothing is mentioned about the origin of crops. The same mistake was made by Gab‐Alla *et al*. (2012), who reported significant differences in organs/body weight and serum biochemistry between rats fed GM and non‐GM maize.

While the bulk of the studies have been on commercialized crops, safety studies on events that were never commercialized have also been widely reported on. Prescott *et al*. (2005) and Sagstad *et al*. (2007) studied the immunogenicity of a GM pea and adverse effects of a GM maize in salmon, respectively. It has not been possible to replicate these studies (Lee *et al*., 2013; Sissener *et al*., 2011). It is now known that pea (both GM and non‐GM) elicit an immunogenic response in mice, and the results obtained in 2005 were not specific to GM pea; for salmon, the effects observed in 2007 corresponded to the effect of confounding factors (i.e. mycotoxins) instead of the GM trait. These cases highlight the importance of repeating experiments in other laboratories to confirm results, before drawing any conclusions.

Fares and El‐Sayed (1998) observed adverse effects when potatoes supplemented with Bt protein—in the form of an uncharacterized crude extract from bacteria—were provided to mice. However, GM potatoes producing Bt protein did not cause significant effects. Purity and concentration of the protein in treated potatoes were not determined, and as it was a crude extract, it has impurities and other proteins unrelated to Bt.

Finally, there are some studies in which the authors remark that their results do not show health concerns, but are nevertheless cited as an example of harm. Kiliç and Akai (2008) found minor histopathological and biochemical effects in rats fed GM maize, but long‐term consumption over three generations did not cause health concerns. Yum *et al*. (2005) recognized several flaws they committed and discussed that they could not conclude GM soya bean is allergenic. Eleven years after this publication, no other studies have reported similar results.

Even well‐designed studies have been misrepresented. Selective omission of details in Dona and Arvanitoyannis (2009) gives the impression of health concerns when there were none (posted at https://pubpeer.com/publications/18989835, accessed 12 March 2017). Séralini *et al*. (2011) used a filtered reference from a large list of papers addressing GM food/feed, safety assessments list in their review, and suggested health concerns from papers in which the original authors concluded no negative effects or concerns on tested animals. For instance, Zhu *et al*. (2004) concluded ‘the results of this 13‐week dietary feeding study demonstrated that the two types of soybean meal prepared from GM herbicide‐tolerant (RR) and nearly isogenic conventional soybeans were comparable in composition and nutritional value for Sprague‐Dawley rats. In addition, there was no evidence of any pathologic signs of RR soybean meal even when included at a high percentage of the diet (two or three times greater than normal)’. However, Séralini *et al*. cited this study pointing out GMO affected body weight increase. Moreover, Séralini *et al*. cited seven of 19 studies of his list as cases where statistical differences were not biologically meaningful for the original authors; however, this can be debated according to his narrative. They did not provide new statistical analysis or details from those articles.

Finally, it is helpful to put these 35 studies in the context of the larger body of literature that has used animal studies to help evaluate food and feed safety. There are at least 204 articles assessing animal health parameters, and 111 studies assessing animal performance, 106 testing nutritional equivalence and 46 addressing allergenicity (Sánchez, 2015). A summary of published data in peer‐reviewed journals comparing feeds from GM plants with their isogenic counterparts and organized by food‐producing animal model can be found elsewhere (Flachowsky and Reuter, 2017).

While the 35 studies showing adverse effects only tested 11 events, the 204 other articles that assess animal health evaluate 94 different events. The soya bean event 40‐3‐2 has been the most analysed (28 studies; 15%), along with the maize event MON810 (25 studies; 14%). In the same subset of papers, different animal models have been used, where rat (46%), mouse (26%), fish (9%), pig (6%) and cow (5%) have been the most common.

At least 44 peer‐reviewed articles describing 90‐day subchronic toxicity feeding studies for nine crops (Ricroch *et al*., 2014) have been published along with 23 long‐term studies and 19 multiple‐generation feeding studies (Ricroch, 2013; Snell *et al*., 2012). No biologically relevant effects on feed intake, digestibility, fertility, performance or animal health have been reported. Furthermore, it has been noted that over 100 billion animals have consumed GM feed with no unfavourable or perturbed trends in animal health and productivity (Van Eenennaam and Young, 2014).

It is always possible that all or some of these studies failed to detect adverse effects simply because animal studies lack enough sensitivity (Bartholomaeus *et al*., 2013). Nevertheless, the point remains that, in contrast to the 35 studies here assessed, when the same events tested have been conducted under a robust study design—that is proper statistical analysis, controls, etc.—no adverse effects have ever been observed.

## Conflict of interest

MAS is employed by ChileBio (www.chilebio.cl), which is funded by companies that develop GM crops. WAP performed public‐sector‐funded research with GM crops and has performed public outreach under the auspices of the ILSI International Food Biotechnology Committee and CropLife International.
